# Cadmium-induced genome-wide DNA methylation changes in growth and oxidative metabolism in *Drosophila melanogaster*

**DOI:** 10.1186/s12864-019-5688-z

**Published:** 2019-05-09

**Authors:** De-Long Guan, Rui-Rui Ding, Xiao-Yu Hu, Xing-Ran Yang, Sheng-Quan Xu, Wei Gu, Min Zhang

**Affiliations:** College of Life Sciences, Shaanxi Normal University, Xi’an, 710062 Shaanxi China

## Abstract

**Background:**

Cadmium (Cd)-containing chemicals can cause serious damage to biological systems. In animals and plants, Cd exposure can lead to metabolic disorders or death. However, for the most part the effects of Cd on specific biological processes are not known. DNA methylation is an important mechanism for the regulation of gene expression. In this study we examined the effects of Cd exposure on global DNA methylation in a living organism by whole-genome bisulfite sequencing (WGBS) using *Drosophila melanogaster* as model.

**Results:**

A total of 71 differentially methylated regions and 63 differentially methylated genes (DMGs) were identified by WGBS. A total of 39 genes were demethylated in the Cd treatment group but not in the control group, whereas 24 showed increased methylation in the former relative to the latter. In most cases, demethylation activated gene expression: genes such as *Cdc42* and *Mekk1* were upregulated as a result of demethylation. There were 37 DMGs that overlapped with differentially expressed genes from the digital expression library including *baz*, *Act5C*, and *ss*, which are associated with development, reproduction, and energy metabolism.

**Conclusions:**

DNA methylation actively regulates the physiological response to heavy metal stress in *Drosophila* in part via activation of apoptosis.

**Electronic supplementary material:**

The online version of this article (10.1186/s12864-019-5688-z) contains supplementary material, which is available to authorized users.

## Background

Cadmium (Cd)-based chemicals are essential in many industries, including plastics and battery manufacturing and non-ferrous metallurgy [1]. As a result of their widespread use, large amounts of Cd have been released into the environment over many decades, causing pollution that threatens global ecosystems as well as human health [2, 3]. Through the food chain, these chemicals can accumulate in organisms inhabiting contaminated environments [4], resulting in genetic damage, reduced reproductive capacity, growth inhibition, and even death [5, 6].

Given their ubiquitous presence, there is an urgent need to better understand the biochemical impacts of Cd-based chemicals and develop effective detoxification mechanisms [7]. Many studies have addressed not only the repair of genetic damage caused by Cd but also apoptosis and oxidative stress [8, 9]. However, there is little known about how Cd affects DNA methylation, a type of epigenetic modification that is important for gene regulation [10–12].

*Drosophila melanogaster* is considered a suitable model species for investigating biological responses to toxic chemicals [13]. Genes in *D. melanogaster* have many homologs in mammals including humans, with many genes being structurally and functionally conserved; however, *Drosophila* has the advantage of a simpler genome that makes it more amenable to studies of complex biological mechanisms [14–16]. Although global DNA methylation level is lower overall in the genome of *Drosophila* as compared to mammals, there are also fewer methylases. DNA methylation is an important epigenetic mechanism for the regulation of gene expression in development, reproduction, and stress resistance [17–20].

Although it is presumed that DNA methylation is involved in the response to Cd stress in *Drosophila*, there have been no detailed surveys of DNA methylation profiles following exposure to heavy metal stress and many questions remain unanswered, including the number and identity of methylated genes and how methylation affects gene expression. To address these points, in this study we used whole-genome bisulfite sequencing (WGBS) to evaluate genome-wide DNA methylation changes in *D. melanogaster* subjected to Cd stress. We identified many differentially methylated genes (DMGs) and demonstrated their relationship to gene expression. Our results provide evidence for the broad involvement of DNA methylation in the response to heavy metal stress in animals.

## Results

### DNA methylation state of the Drosophila genome

WGBS yielded 35.5 Gb of raw data from six different samples (three repeats for each of the two groups) comprising about 38.2 billion nucleotides, all with Q20 values above 95% (Table 1). The raw reads numbered more than 37.6 million among the six samples, and after removing those of low quality (i.e., those with a high number of ‘N’, poly-A contamination, and contamination by adaptor sequences), at least 98% of the reads were retained and were taken as the high-quality (HQ) clean reads. Given the number of retained HQ reads, we expected an average genome coverage of about 30×. For all samples, between 63.56 and 74.60% of the HQ reads mapped uniquely to the genome, giving an average genome coverage between 27.28× and 35.67× (Table 1).

**Table 1:** Summary of genome-wide bisulfite sequencing data for six *Drosophila melanogaster* samples

Sample	Clean data (bp)	HQ clean data (bp)	No. of clean read	No. of HQ clean reads (%)	Q20 (%)	Q30 (%)	GC (%)	N (%)	HQ clean data / clean data (%)
CK-1	6,342,804,900	6,265,486,794	42,285,366	41,829,038 (98.92%)	6,127,259,145 (97.79%)	5,943,780,984 (94.87%)	1,182,385,554 (18.87%)	987,865 (0.02%)	98.78%
CK-2	5,651,708,700	5,543,296,390	37,678,058	36,998,402 (98.20%)	5,328,517,738 (96.13%)	5,021,173,126 (90.58%)	1,040,523,808 (18.77%)	593,380 (0.01%)	98.08%
CK-3	6,667,975,500	6,566,612,690	44,453,170	43,845,552 (98.63%)	6,346,014,536 (96.64%)	6,013,608,683 (91.58%)	1,291,540,988 (19.67%)	715,638 (0.01%)	98.48%
s52–1	6,380,107,200	6,289,532,315	42,534,048	41,983,774 (98.71%)	6,135,594,846 (97.55%)	5,933,616,057 (94.34%)	1,179,393,128 (18.75%)	976,796 (0.02%)	98.58%
s52–2	6,989,049,900	6,860,524,362	46,593,666	45,809,874 (98.32%)	6,685,079,771 (97.44%)	6,464,628,728 (94.23%)	1,303,391,516 (19.00%)	1,073,466 (0.02%)	98.16%
s52–3	6,172,341,000	6,053,568,099	41,148,940	40,413,472 (98.21%)	5,787,545,449 (95.61%)	5,425,219,375 (89.62%)	1,168,998,770 (19.31%)	567,912 (0.01%)	98.08%

*HQ* high quality

**Fig. 1:**
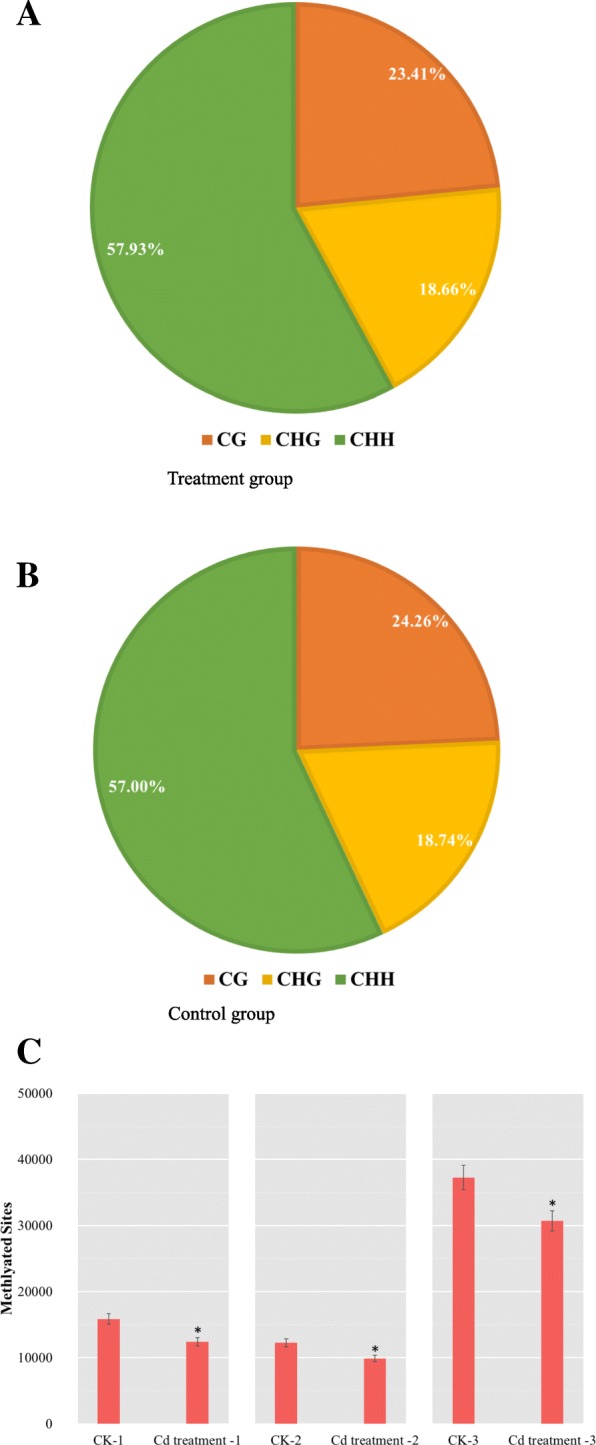
Distribution of mC in CG, CHG, and CHH in the (**a**) treatment group, (**b**) control group and (**c**) all six different samples

The average number of methylated cytosines detected in the Cd treatment and control groups was about 0.1% of all cytosines in the *Drosophila* genome. There were 12,397 methylated cytosines for CG, 9880 for CHG, and 30,678 for CHH (where H represents A, C, or T) in the treatment group (Fig. 1a and Table 2), which was significantly lower (*P* < 0.05, Fisher’s exact test) than in the control group (15,854, 12,243, and 37,246, respectively, Fig. 1b and Fig. 1c), indicating that Cd treatment reduced global methylation levels.

**Table 2:** Methylated CG, CHG, and CHH sites in Cd treatment and control groups (CK) as the total number and percentage of whole genome

Group	Type	Number	Percent (%)
CK-1	CG	15,854	24.26
CK-2	CHG	12,243	18.74
CK-3	CHH	37,246	57.00
Cd treatment 1	CG	12,397	23.41
Cd treatment 2	CHG	9880	18.66
Cd treatment 3	CHH	30,678	57.93

### Preferred sequences flanking the methylation site

**Fig. 2:**
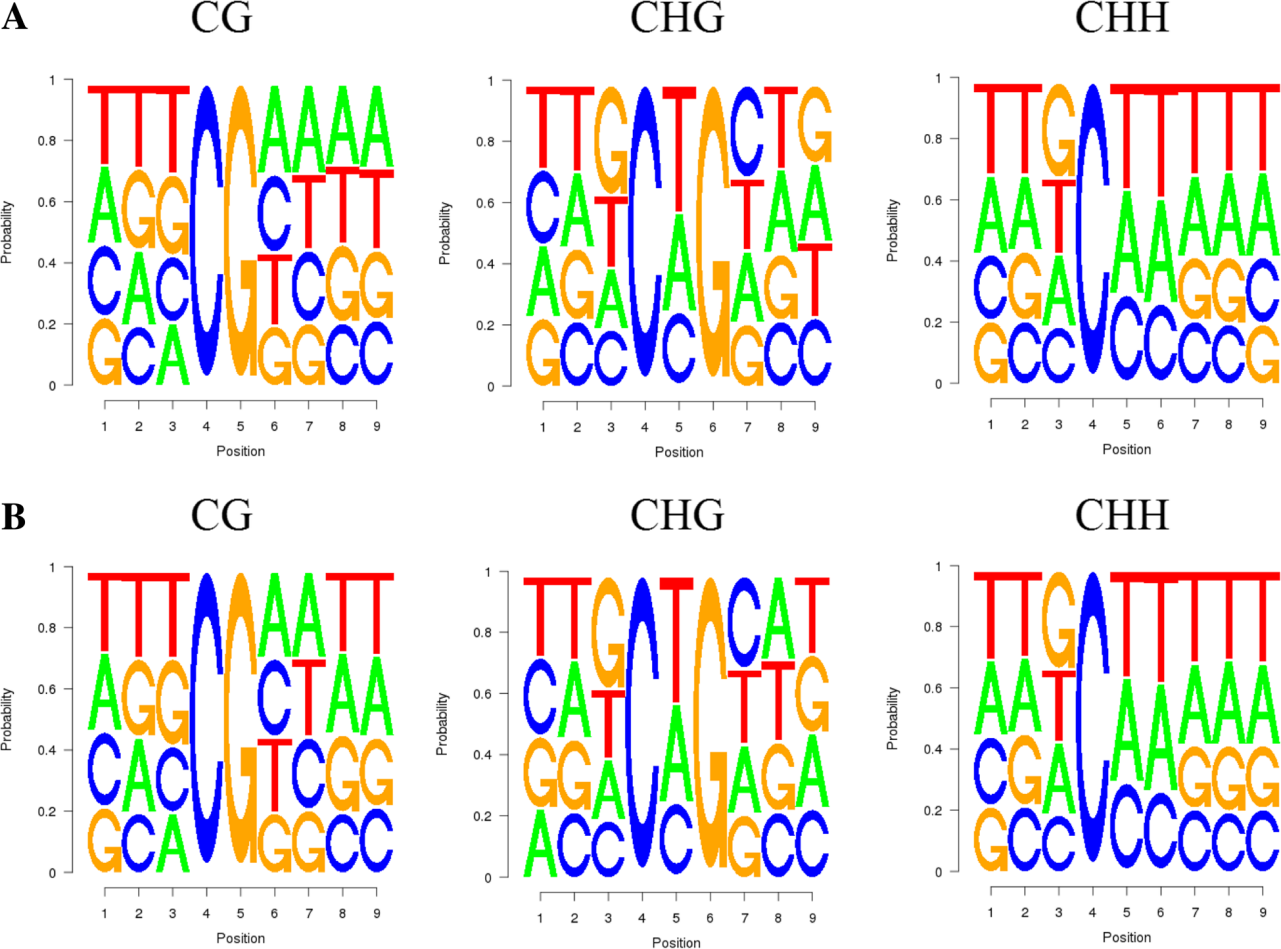
Sequence preferences of methylation site domains in CG, CHG, CHH in (**a**) treatment group and (**b**) control group. The x axis represent the 9-mer sequence while the y axis represent the probability of each type of nucleotide

We analyzed the relationship between the type of methylation and surrounding sequences by identifying the features of the 9-mer sequence around the methylation site (Fig. 2a and b). For CHH, the Cd treatment and control groups showed identical sequence enrichment at each genomic region, with “TTG” and “TTT” being the preferred sequences around the methylation site. In the CG and CHG environment, sequences around methylation were slightly different. At the CG locus, with “TTT” and “AAAA” being the preferred sequences of the treatment group and “TTT” and “AATT” being those of the control group. Judging by such pattern, there seem to be almost equal preference for A, T, C, and G around all types of methylation sites. Thus, there doesn’t seem to be any significantly enriched motifs in any of the treatments. Methylation occurred at similar sequence environments.

### DNA methylation levels in different genomic regions

**Fig. 3:**
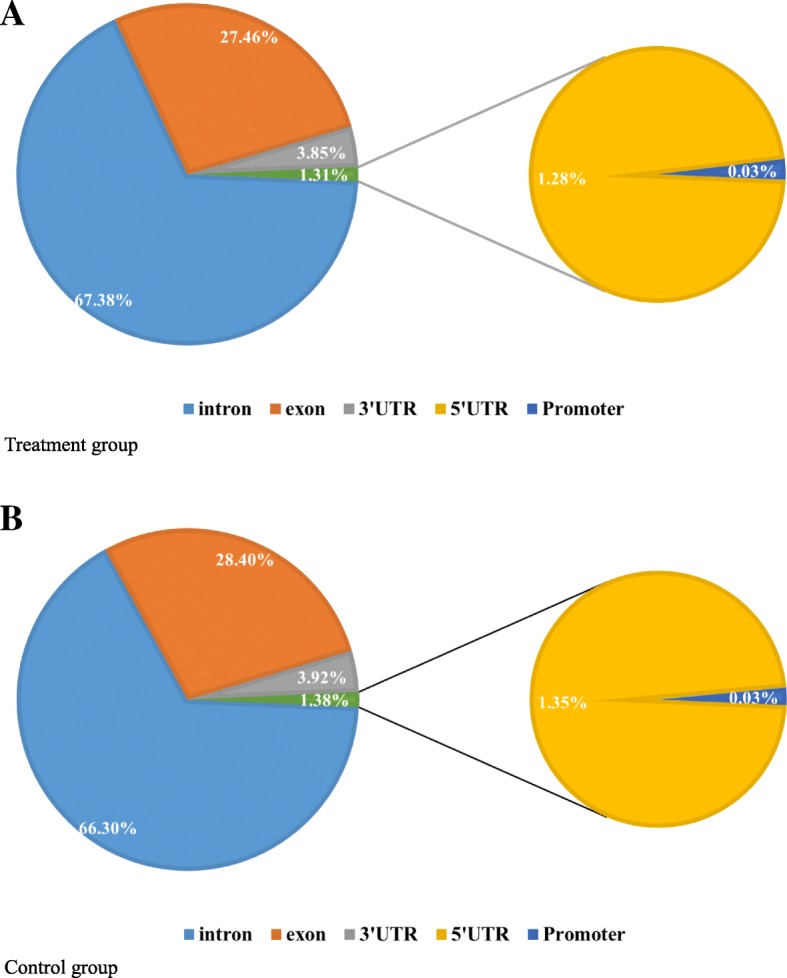
Distribution of different methylation types

**Fig. 4:**
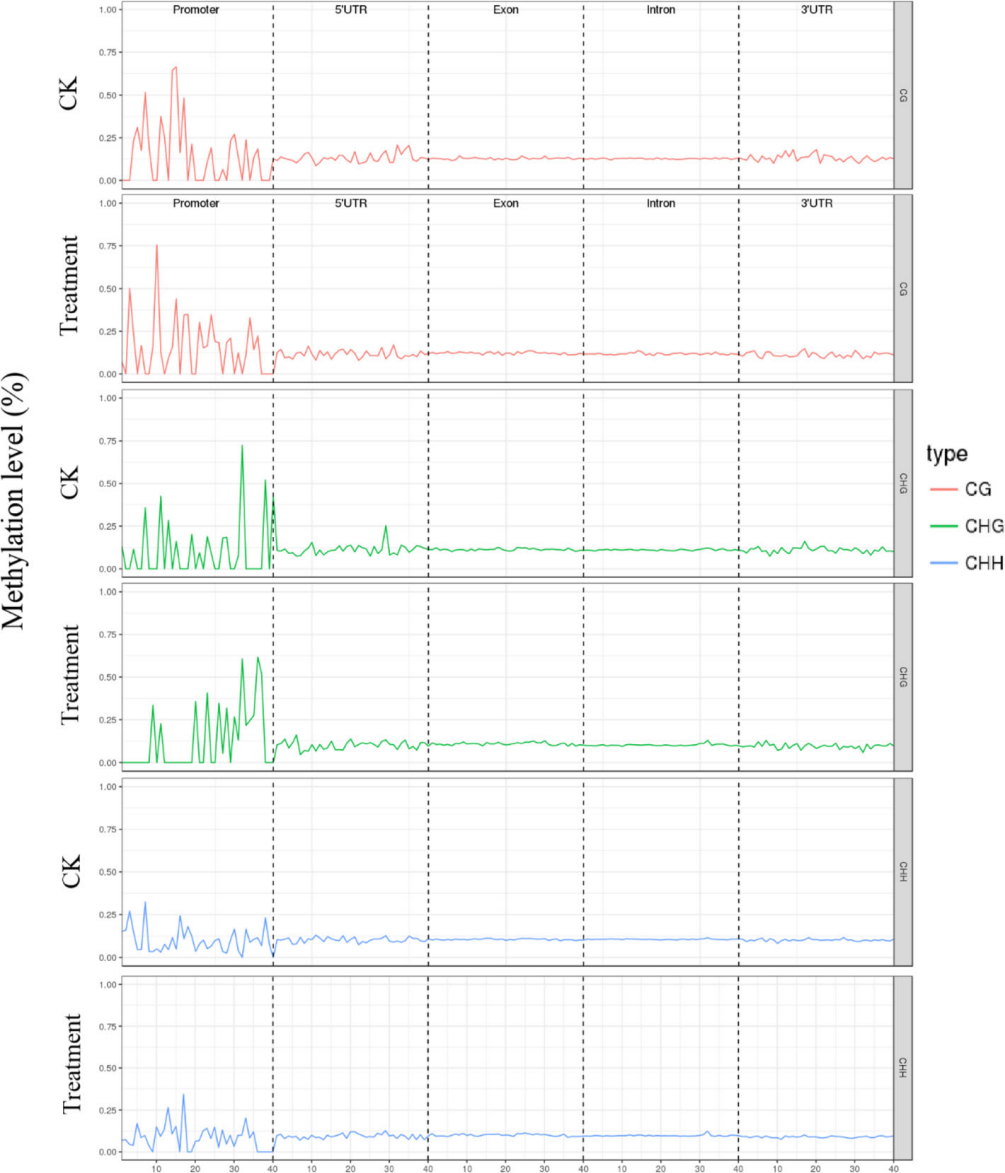
DNA methylation levels in different functional regions of Cd treatment and control groups. The coordinates are compressed according to the size of the region, while the x-axis represents the positions of different regions, and the y-axis represents the level of methylation

DNA methylation levels generally show a varied distribution across different functional regions of the genome. We examined the distribution of DNA methylation sites and found that the methylation levels in the promoter, 5′ untranslated region (UTR), exon, intron, and 3′ UTR were similar between Cd treatment and control groups (Fig. 3). The promoter region had the fewest methylation sites (0.03% of all sites), whereas those in introns accounted for over 65% of total sites (Fig. 3a, b). We used the sliding window method to examine DNA methylation levels in these five gene components. Methylation levels were similarly distributed in the treatment and control groups (Fig. 4). Compared to other genetic components, changes in methylation level were observed in the promoter region, but the overall methylation level was high. Methylation levels did not differ significantly across regions, and there was little change in the exon and intron, which showed a stable distribution of methylation marks.

### DMRs and related genes

We used swDMR software with stringent parameters to identify DMRs between Cd treatment and control groups. The methlytion signals, along with QQ-plots of *P* values associated with the DMRs, and the range of *P* values are shown in Additional file 1: Figure S1, Additional file 2: Figure S2 and Additional file 3: Figure S3. The QQ-plots shows that all dots represent observed log *p*-values of CG\CHG\CHG formed an almost straight line that away from the line that represent log p-values under the null expections, which indicate these DMRs are actually siginificate deviated. A total of 71 DMRs were detected throughout the genome (Additonal file 4 Table S1). To identify the methylated genes, we used the genomic localization of each DMR and information on *D. melanogaster* genome structure annotation to label the DMRs, and determined that they belong to 63 genes (Additional file 1: Table S1).

**Fig. 5:**
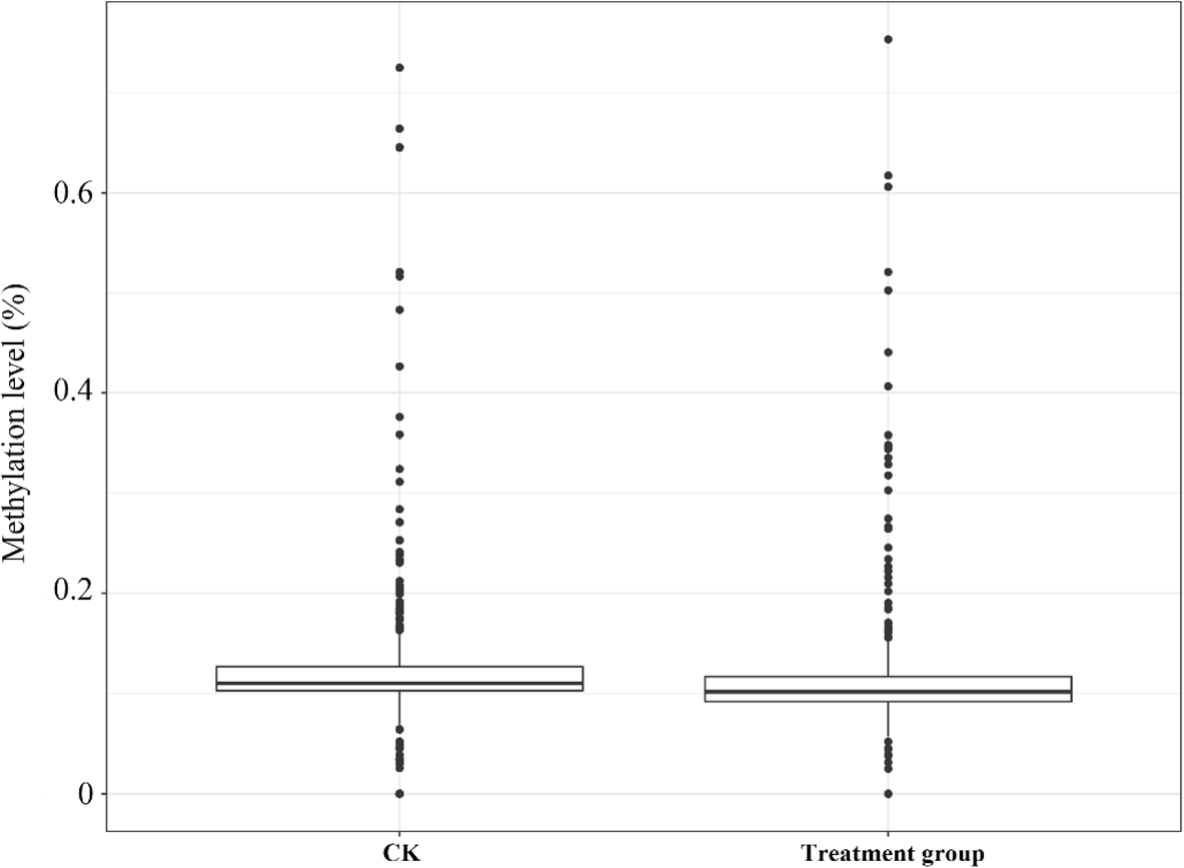
Methylation levels of DMRs in the Cd treatment and control groups. The box plot shows 25–75% quartiles; the black line in the box represents the median distribution (50% quartile)

**Fig. 6:**
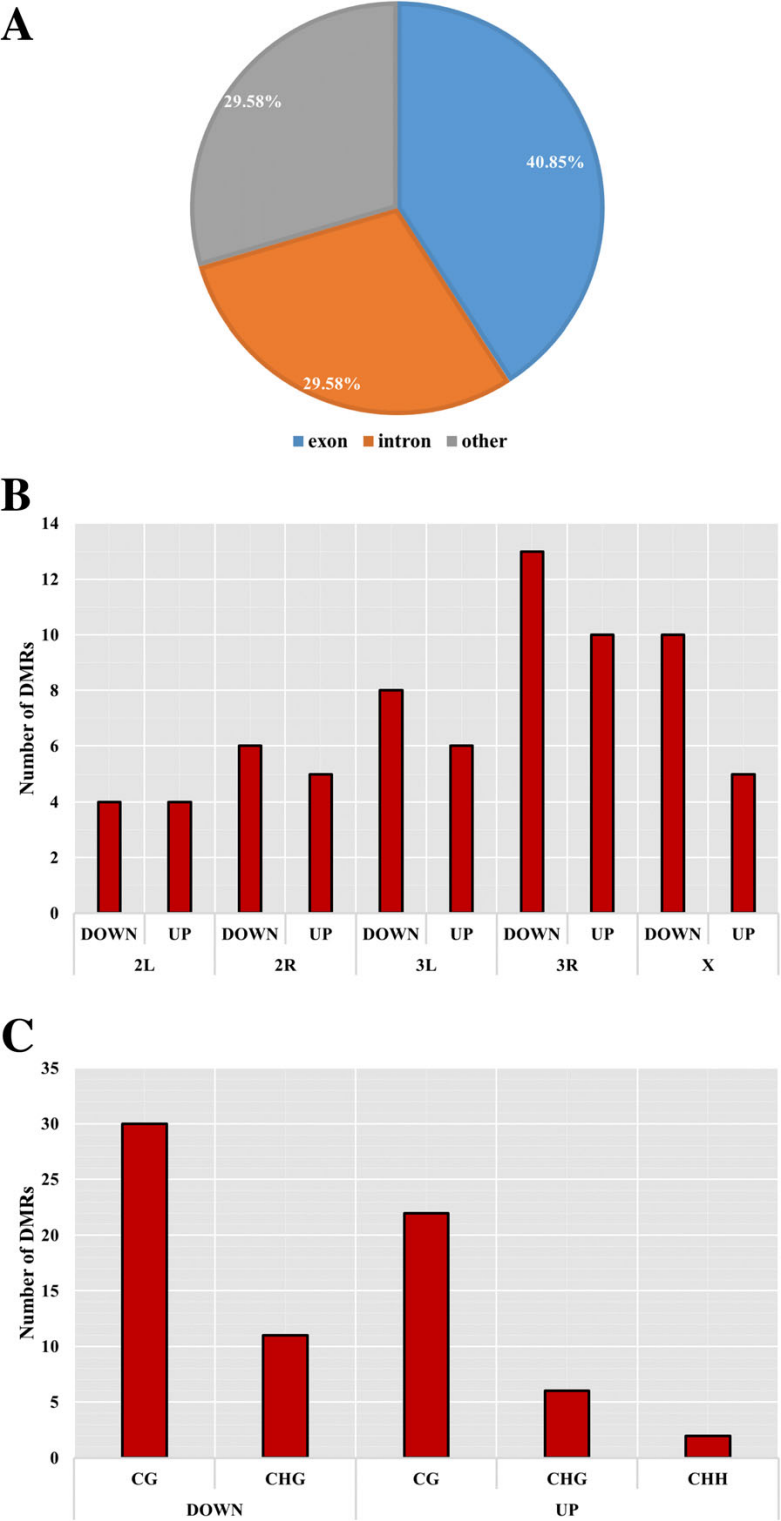
Distribution statistics for differential DMRs. The different graphs show the distinct features of DMRs. **a** Results of segmentation using different functional regions. **b** Results of chromosome location discrimination. **c** Discrimination results using different methylation types

In the treatment group, 30 DMRs in 24 genes were hypermethylated and 41 DMRs in 39 genes were demethylated relative to the control group. Thus, the rate of demethylation was greater than the rate of hypermethylation. A box plot analysis of the DMRs showed that the methylation level was slightly lower in the treatment as compared to the control group (Fig. 5), indicating that in addition to the number of de−/hypermethylated sites, demethylation occurred at a higher rate during Cd exposure. Moreover, the DMRs were mainly distributed in introns and exons—i.e., 21 and 29, respectively (Fig. 6a), with most located on chromosome 3R, followed by chromosomes 3 L and X. With the exception of chromosome 2 L, hypermethylation was less frequently observed than demethylation on all chromosomes, with chromosome 2R having the lowest level of demethylation (Fig. 6b). Additionally, more DMRs were demethylated than were methylated, and methylation sites of the CHH type were mostly demethylated (Fig. 6c).

### Gene ontology (GO) and Kyoto encyclopedia of genes and genomes (KEGG) enrichment analyses of DMRs

**Fig. 7:**
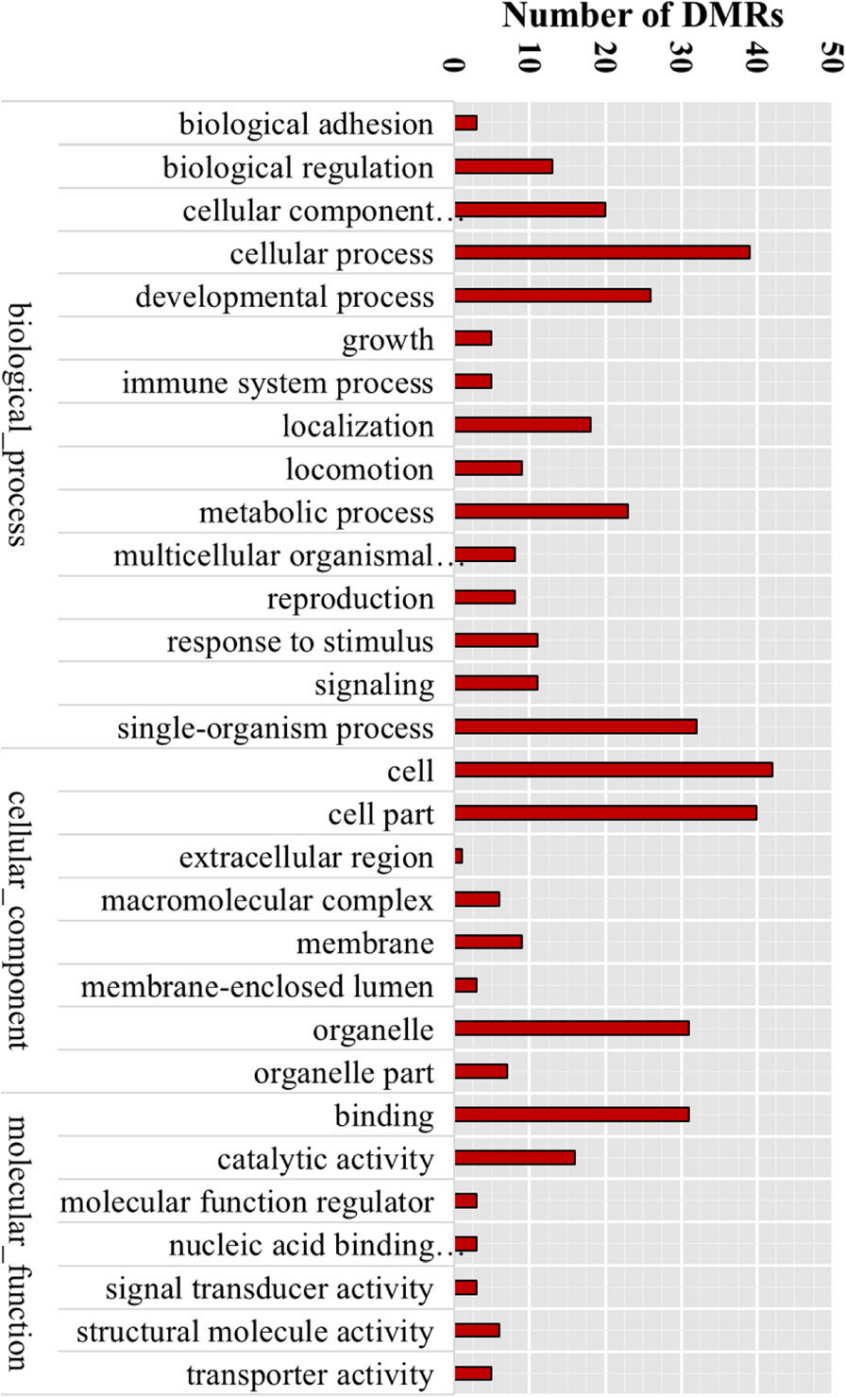
GO terms of enriched DMRs. Bars represent the number of the DMRs in enriched GO terms

We carried out GO and KEGG enrichment analyses for all DMRs to clarify the functional significance of differential methylation (Additional file 4: Table S1). For all DMRs, the enriched GO terms were related to critical biological processes in *D. melanogaster* including reproduction, locomotion, development, growth, and response to stimulus, indicating that Cd exposure affects the methylation of genes related to the basic physiology of *Drosophila* (Fig. 7 and Additional file 5: Table S2). This was true for both hyper- and demethylation, suggesting that DNA methylation broadly affects gene regulation in intricately connected biological processes. We also found other GO terms that were enriched such as immune system, single-organism process, and biological regulation. In the cell component and molecular function categories, organelle and catalytic activity were significantly enriched. Thus, genes regulated by DNA methylation are not limited to those directly involved in the response to Cd toxicity; instead, epigenetic modifications are associated with overall regulation of gene expression.

**Fig. 8:**
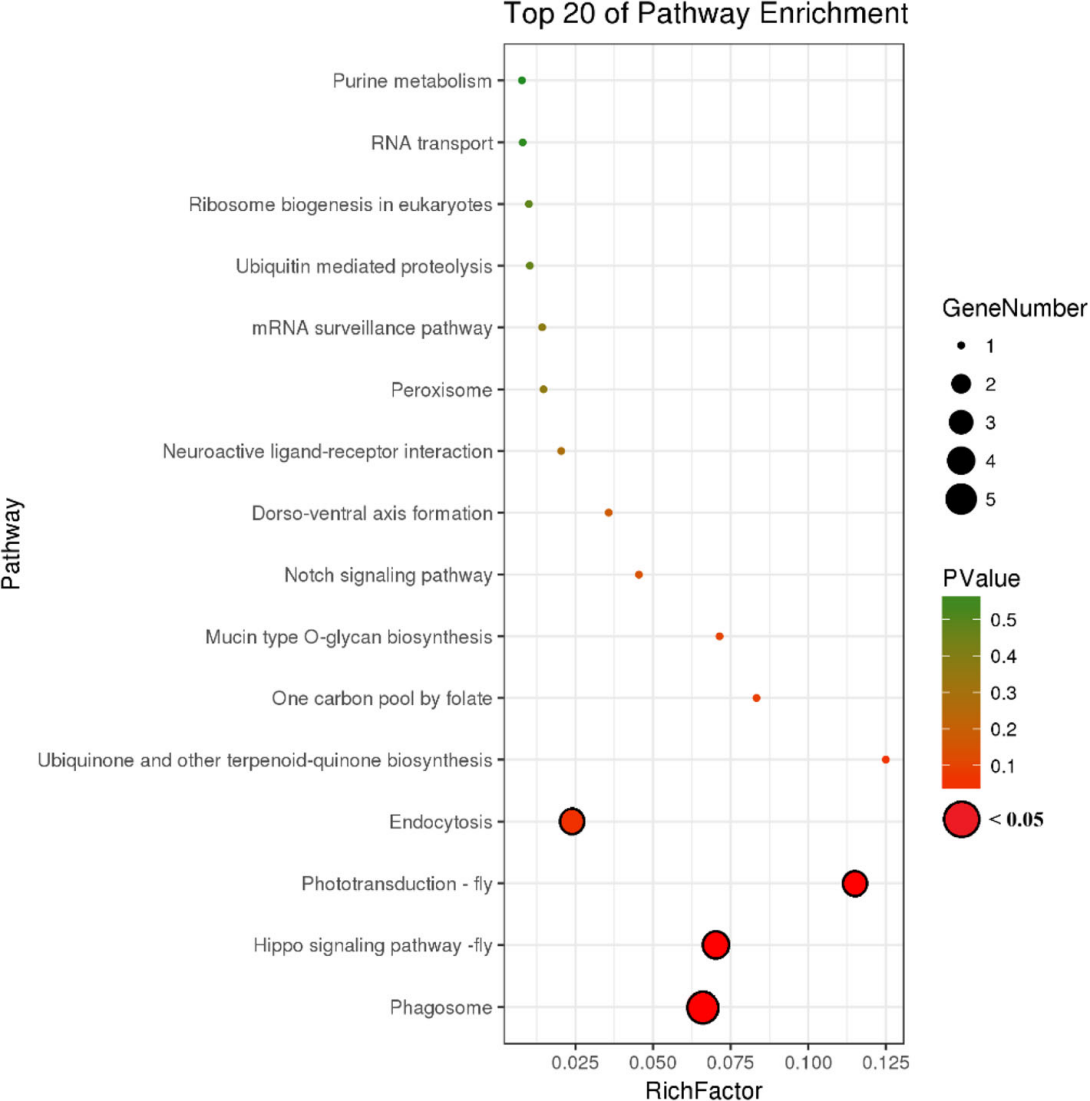
KEGG terms of enriched DMRs. *P* values and gene numbers are represented by circles of different colors and size

Cd exposure altered a variety of pathways in the KEGG enrichment analysis; the top pathways are shown in Fig. 8, and included phagosome, phototransduction, and Hippo and Notch signaling pathways (Additional file 5 Table S2). In agreement with the enriched terms identified by GO analysis, these pathways are associated with reproduction and development. Thus, the results of the KEGG analysis demonstrate that multiple cellular mechanisms are activated in the response to Cd exposure and that DNA methylation is actively involved in their regulation.

### Association between DMGs and differentially expressed genes (DEGs)

**Fig. 9:**
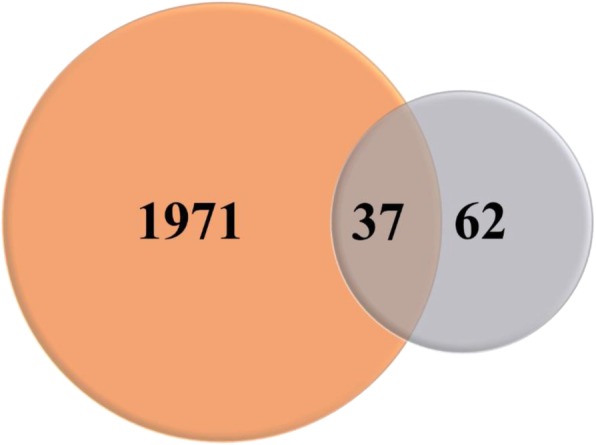
Number of DMG and DEG overlaps between Cd treatment and control groups. DMG and DEG are represented as orange and gray, respectively; overlapping regions are DMGs with differential expression

The GO and KEGG enrichment analyses of DMRs provided insight into the processes affected by DNA methylation in *D. melanogaster* in response to Cd treatment on a large scale. To identify the specific genes involved in these processes, we compared the complete gene sequences of these DMRs—that is, DMGs—with *Drosophila* digital expression library (DGE) data obtained under the same Cd treatment conditions as those of the present study. In aim to test whether these overlaps are meaningful, same number of genes and genomic regions were randomly picked and counted for the overlap for 25 times. When we are sure about that the random overlap will not likely to affect the results, we finally identified 1971 DEGs associated with heavy metal Cd stress in *D. melanogaster*, of which 37 were associated with 62 DMRs (Fig. 9 and Additional file 6: Table S3). This represented only a small proportion of all DEGs (1.87%); on the other hand, the fraction of DMGs was very high (59.6%), indicating that changes in DNA methylation state regulate gene expression but are not the main regulatory process in *Drosophila*. The observed correspondence between methylation and gene expression levels provide further evidence that DNA methylation regulates gene expression in combination with other mechanisms, and may only occur at specific sites in genes. In most of the 37 DMRs, methylation levels were negatively correlated with gene expression level, that is, methylation repressed gene expression, which in turn activated expression. Exceptions to this trend include *Eip75B*, a gene related to female gamete production whose expression increased with methylation level.

**Fig. 10:**
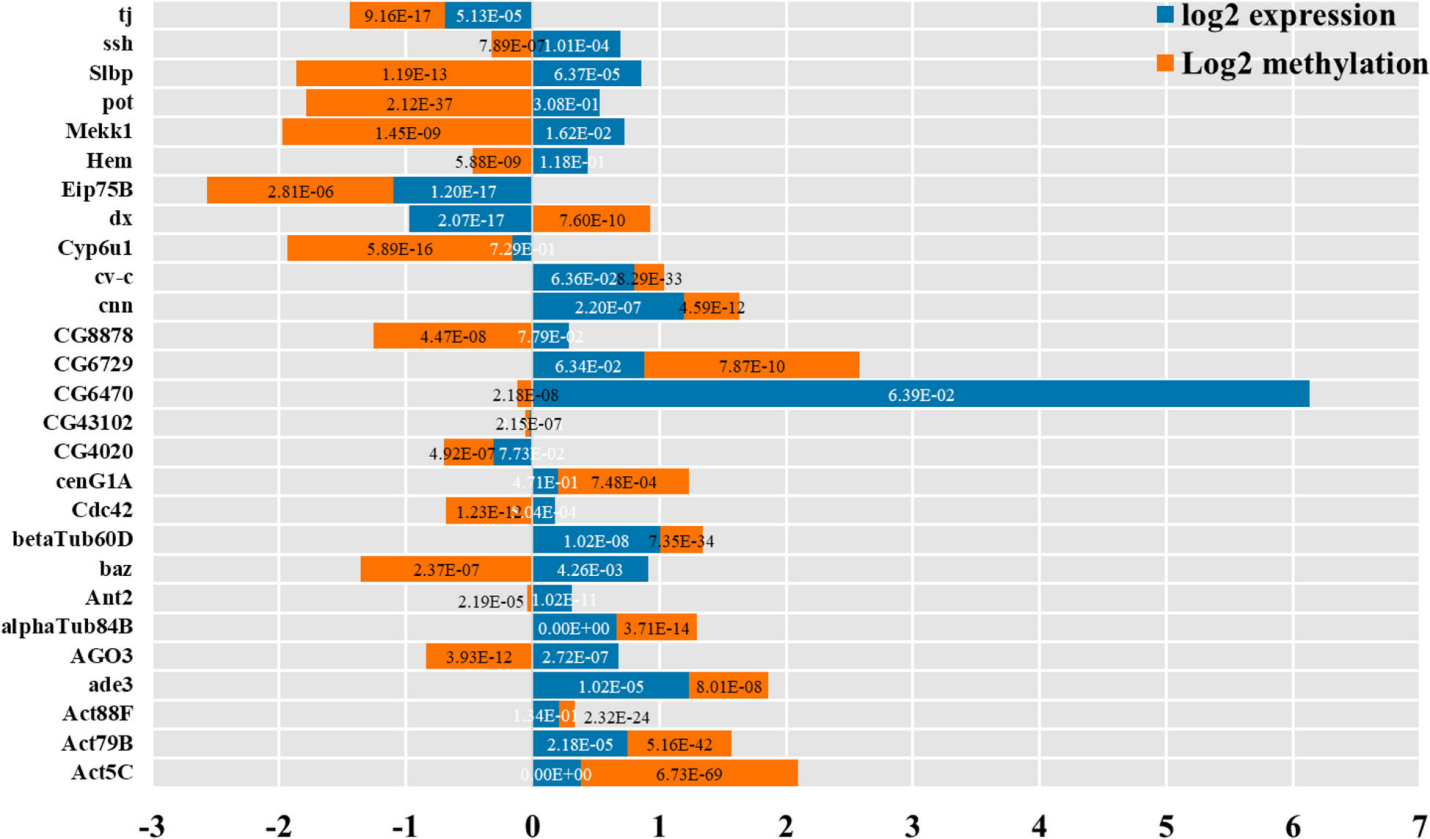
Methylation and expression levels of 27 candidate genes that are critical for the response to Cd stress. Changes in methylation (orange bars) and expression (blue bars) are shown. Values were calculated for the Cd treatment vs. the control group. The depth of methylation and transcripts per million were used in the calculation and log2 analysis was used to standardize the data. Values less and greater than zero represent down- and upregulated genes, respectively. *P* values are shown for each fold change; those in white and black fonts were calculated based on methylation and expression fold change, respectively

An analysis of the 37 overlapping genes showed that 27 of these had critical functions (Fig. 10) that were related to development and reproduction according to the GO and KEGG pathway analyses, including *cnn*, *ssh*, *Act5C*, *pot*, *baz*, *Cdc42*, *Hem*, *Eip75B*, and *cv-c*. We also found four genes (*ade3*, *CG6729*, *Slbp*, and *CG8878*) related to metabolic biosynthesis and 13 involved in resistance to Cd stress (*cenG1A*, *Cyp6u1*, *AGO3*, *betaTub60D*, *alphaTub84B*, *Act79B*, *Act88F*, *CG43102*, *dx*, *Ant2*, *CG6470*, *Mekk1*, *CG4020*, and *Cdc42*). These genes have binding or transferase activity and are associated with the immune system or intracellular signaling pathways, with functions in antioxidant and metal ion binding as well as resistance to external stimuli and initiation of apoptosis. Of these genes, *Mekk1* has been linked to the response to Cd toxicity through positive regulation of the mitogen-activated protein kinase (MAPK) cascade, whereas *Cdc42* is closely related to cell cycle arrest and apoptosis.

We focused on 12 genes for which there was a negative correlation between changes in methylation and expression patterns—namely, *dx*, *Cdc42*, *CG8878*, *Ant2*, *Hem*, *pot*, *AGO3*, *ssh*, *Mekk1*, *Slbp*, *baz*, and *CG6470* (Fig. 10). The expression levels of these genes were upregulated (except for *dx*, which was downregulated) in response to Cd stress through DNA methylation.

## Discussion

DNA methylation is an epigenetic regulatory mechanism that controls gene expression through modification of cytosine bases that alters chromatin structure and stability and DNA–protein interactions [21, 22]. There is increasing evidence that DNA methylation is a mechanism in animals that allows adaptation to environmental stress or trauma [23] through controlled changes in gene expression levels [24–27].

In this study, we determined that Cd ion stress altered DNA methylation patterns in the *Drosophila* genome by WGBS. Although the DNA methylation rate in the genome was very low (~ 0.1%), it affected genes related to the stress response to Cd exposure. We also identified 71 DMRs encompassing 63 genes. In general, demethylation of genes in these regions was associated with increased gene expression in response to Cd treatment, which is contrary to previous findings that DNA methylation has a strictly inhibitory role in transcription [28, 29]. It is worth noting that the demethylated genes had functions associated with essential biological processes such as development, reproduction, cellular defense and repair, antioxidant stress, and apoptosis.

Detoxification proteins are continuously synthesized in cells exposed to toxic elements. The results of our study suggest that this is regulated by DNA demethylation in *Drosophila* exposed to the heavy metal Cd, leading to the activation of stress resistance genes. Our results provide new evidence for the biological importance of DNA methylation and insight into how gene expression is regulated by epigenetic modifications under conditions of stress.

GO and KEGG enrichment analyses can be used to analyze the functions of DEGs [30]. In this study, we carried out a functional enrichment analysis of GO terms and KEGG pathways for all DMRs [31, 32] and found that DNA methylation during Cd stress affects genes that are involved in basic physiological functions. Enriched GO terms included reproduction, locomotion, development process, growth, immune system, and response to stimulus. This is in agreement with previous reports that Cd inhibits development and leads to decreased fertility [33] and reduced immunity [34, 35]. Similar results were obtained by KEGG pathway analysis, which identified pathways associated with the phagosome, Hippo and Notch signaling, and phototransduction as those affected by changes in DNA methylation as a result of Cd stress; these processes and pathways are implicated in the regulation of immunity, somite development, ocular development, neurogenesis, and embryogenesis. Thus, DNA methylation can directly affect biological mechanisms such as development and immunity to counteract Cd toxicity, which has not yet been demonstrated; most previous studies have suggested that the mechanism of resistance to Cd stress in vivo is indirect, involving free radical scavenging (e.g., glutathione, heat shock protein, and metallothionein) or apoptosis.

We examined genes showing the greatest differences in expression due to changes in DNA methylation [36] and identified 27 including *AGO3*, *Myo81F*, and *Cdc42* from the set of 37 DMGs overlapping with the DGEs. These 27 genes covered all the biological mechanisms identified by GO and KEGG enrichment analyses; 12 showed changes in methylation that were consistent with the change in their expression level, while 11 were upregulated as a result of demethylation following Cd treatment.

Previous studies have demonstrated that DNA methylation is implicated in development and reproduction [37, 38]. Two DMGs in this study—namely, *ssh* and *Act5C*—are involved in eye and brain development, respectively. In addition, *baz*, *Eip75B*, *cv-c*, and *cnn*—which are involved in oocyte axis specification, female gamete generation, embryonic morphogenesis, and embryo development, respectively—were also differentially methylated, indicating that epigenetic regulation of genes involved in resistance to heavy metal toxicity begins when the fertilized egg begins to form and is passed on to offspring.

We also identified four DMGs related to metabolic biosynthesis, namely *ade3*, *CG6729*, *Slbp*, and *CG8878*. Although the methylation patterns of these genes was inconsistent, all showed increased expression. These four genes are associated with purine nucleotide metabolism, nuclear-transcribed mRNA catabolism, histone mRNA metabolism, and protein modification, and their upregulation implied that Cd exposure induced base utilization, mRNA recovery, and protein synthesis rate and consequently, gene transcription and protein translation in *Drosophila*. This expression profile is consistent with the mechanism of stress resistance and demonstrates that it is not possible to predict changes in the regulation of gene expression based solely on changes in DNA methylation levels.

The most important findings of this study are that we identified eight genes related to the immune system and intracellular signaling that were differentially methylated by Cd treatment (*Cdc42*, *cenG1A*, *CG43102*, *Mekk1*, *betaTub60D*, *alphaTub84B*, *Act79B*, and *Act88F*). These genes are implicated in cell death or apoptosis and their methylation has been linked to a variety of malignancies and metabolic diseases [39, 40]. Apoptosis can be activated by stressful stimuli such as Cd exposure and determines cell fate in organisms [41, 42]. On the other hand, *alphaTub84B* and *Act79B* are involved in regulation of the actin cytoskeleton and mitotic spindle. *Mekk1* is a previously reported Cd stress resistance gene encoding a zinc finger protein that binds Cd ions and induces mitotic arrest, triggering apoptosis. *Cdc42* (also known as *cenG1A* or *DG43102*) is a well-known gene associated with cancer cell proliferation [43, 44] belonging to the Rho family of small GTPases that regulate mitosis, establishment of cell polarity [45], cell migration [46], and MAPK signaling [47]. Upregulation of *Cdc42* is a marker for cellular responses to external stimuli; in this study, we found that *Cdc42* was demethylated, which corresponded to an increase in gene expression.

In conclusion, the results of this study demonstrate that changes in DNA methylation in *Drosophila* caused by exposure to Cd activate genes involved in apoptosis and other basic cellular processes. These findings provide novel insight into physiological response to heavy metal stress in a multicellular organism and a basis for the development of measures to alleviate the effects of these toxic compounds in humans and other animals.

## Methods

### Experimental Drosophila

The *D. melanogaster* line used in this study was maintained at the Institute of Genetics, School of Life Sciences, Shaanxi Normal University. The strain was from the University of Cambridge (Cambridge, UK) and is guaranteed to have a consistent genetic background.

### Cd treatment

Adult female flies were exposed to Cd at a concentration of 52 mg l^− 1^ and maintained on standard *Drosophila* cornmeal-sucrose-agar-yeast medium at 25 °C ± 1 °C on a 12:12-h light/dark cycle for 10 days.

### Genomic DNA extraction

Genomic DNA was extracted from samples comprising approximately 50 *Drosophila* whole muscle tissues using the DNeasy Blood & Tissue kit (Qiagen, Hilden, Germany) according to the manufacturer’s instructions. Genomic DNA contamination and degradation were verified by agarose gel electrophoresis [48, 49]. DNA purity (OD260/280 ratio) was measured using a NanoPhotometer spectrophotometer (Implen, München, Germany) and DNA concentration was measured using a Qubit 2.0 fluorometer (Life Technologies, Carlsbad, CA, USA) [50, 51].

### Database preparation and quantification

The isolated genomic DNA (5 μg) was used to construct a library for the Cd treatment and control groups. The DNA was sonicated to 200- to 300-bp fragments using a S220 sonicator (Covaris, Woburn, MA). After purification, the DNA fragments were end-repaired, with an adenine added to the two 3′ termini. The cytosine methylation sequencing linker was then ligated to both ends of the DNA fragments, which were treated with the EZ DNA Methylation-Gold kit (Zymo Research, Irvine, CA, USA) and bisulfite and PCR amplified. The length of the insert was assessed using an Agilent Bioanalyzer 2100 system with a Qubit 2.0 fluorometer and library concentration was determined by quantitative PCR. The library was subjected to two-terminal sequencing of 125-bp fragments using the HiSeq 2500 platform (Illumina, San Diego, CA, USA).

The image data were converted to the original sequence (sequencing reads) by base calling and were stored as a FASTQ file. The sequencing error rate and substrate content distribution along each read were analyzed with internal Perl scripts. The original reads in FastQ format were processed using Trimmomatic software by (1) removing the joint; (2) rejecting > 10% of reads containing N (unknown substrate); and (3) removing low-quality reads (low quality score with Phred score ≤ 20). At the same time, the Q20, Q30, and GC contents of the data were calculated using internal scripts.

### Read mapping to the reference genome

After filtering low-quality reads, bisulfite-treated reads were aligned to the downloaded *D. melanogaster* genome sequence using Bismark v.0.12.5 software with default parameters (NCBI, *ftp://ftp.ncbi.nih.gov/genomes/drosophila* *melanogaster*). Prior to mapping, the *D. melanogaster* genome sequence and post-treatment reads were converted to a bisulfite-transformed version (C-to-T and G-to-A conversion), and the transformed genome sequences were indexed using Bowtie2 software (http://bowtie-bio.sourceforge.net/bowtie2/index.shtml). Reads that were perfectly matched from the forward and reverse sequencing data were retained for further mapping against the reference *Drosophila* genome.

The methylation status of all cytosine positions in the reads was inferred from the mapping results. Identical reads that were aligned to the same location in the *Drosophila* genome were considered as replicate reads; these were used to summarize the sequencing depth and coverage of each sample. The results of the methylated extract were converted to bigWig format so that they could be viewed using the IGV browser. The unconverted sodium bisulfite was determined as the percentage of sequenced cytosines that were sequenced at the reference position in the phage genome.

### DMR analysis

Based on the methylation information for each site, DMRs were confirmed using swDMR software [7, 8]. The genomes of the treatment and control groups were first scanned using the sliding window method with a window size of 1000 bp and step size of 100 bp. Only windows containing more than 10 cytosine sites were retained and used to calculate the average methylation level. Those with a fold change (i.e., a difference in average methylation level) between the two samples of > 2 and > 0.1 and a *P* value < 0.05 (reflecting a significant change) with Fisher’s exact probabilistic test were considered as potential DMRs. The above procedure was repeated until the genomes of all potential DMRs were confirmed, and their *P* values were corrected by the false discovery rate method (corrected *P* < 0.05). Thereafter, overlapping potential DMRs were subjected to one-time merging and statistical analysis, and the final merged tends were considered as alternative DMRs (Additional file 4: Table S1).

We compared the chromosomal location information of the DMR with the standard gene file of the *D. melanogaster* reference genome. When a DMR overlapped with a gene or functional component of a gene (such as the 5′ or 3′ UTR, exon, or intron), it was assigned to that gene and its components. The 5′ and 3′ UTR, exon, and intron positional information for each gene was obtained from the standard gene file. The promoter region contained a 2-kb region upstream of the transcription start site.

### GO and KEGG enrichment analysis of DMGs

We used GOseq R package to perform GO and KEGG enrichment analyses of DMGs [52, 53]. Using the hypergeometric distribution test, a corrected P value below 0.05 was set to identify DMRs significantly enriched for GO terms. KEGG pathway enrichment analysis was carried out using the whole genome as background to calculate the significance; according to the background, enriched metabolic pathways were identified based on the number of DMGs. The *P*-values of these GO and KEGG terms were shown in Additional file 5: Table S2.

## Additional files

Additional file 1: **Figure S1.** Methylation level of each DMR (all 71 were included) among six samples. The x and y axes show different samples and their overall methylation levels, respectively. (DOCX 312 kb)

Additional file 2: **Figure S2.** QQ-plots of all the observed log *p*-values VS the expected log p-values under the null expections of CG (mCG), CHG (mCHG), and CHH (mCHH) sites, respectively. (DOCX 99 kb)

Additional file 3: **Figure S3.** Box plot of *P* values among all detected methylated nucleotide sites. X and Y axes show different chromosomes and *P* values, respectively. (DOCX 89 kb)

Additional file 4: **Table S1.** Genes associated with identified DMRs and DMGs. (XLSX 12 kb)

Additional file 5: **Table S2.** Descriptions of enriched GO terms and KEGG pathways. (XLSX 12 kb)

Additional file 6: **Table S3.** Gene names and annotations appearing in the lists of DMGs as well as DEGs. (XLSX 15 kb)

## Abbreviations

DMG: Differentially methylated gene
DMR: Differentially methylated region
GO: Gene Ontology
KEGG: Kyoto Encyclopedia of Genes and Genomes
WGBS: Whole-genome bisulfite sequencing.

## References

[1] Meharg AA, Norton G, Deacon C, Williams P, Adomako EE, Price A, Zhu Y, Li G, Zhao FJ, Mcgrath S (2013). Variation in rice cadmium related to human exposure. Environ Sci Technol.

[2] Pedersen KL, Bach LT, Bjerregaard P (2014). Amount and metal composition of midgut gland metallothionein in shore crabs (Carcinus maenas) after exposure to cadmium in the food. Aquat Toxicol.

[3] Song NH, Koh JW (2012). Effects of cadmium chloride on the cultured human lens epithelial cells. Mol Vis.

[4] Ding P, Zhuang P, Li Z, Xia H, Lu H (2013). Accumulation and detoxification of cadmium by larvae of Prodenia litura (Lepidoptera: Noctuidae) feeding on cd-enriched amaranth leaves. Chemosphere.

[5] Cabral M, Toure A, Garçon G, Diop C, Bouhsina S, Dewaele D, Cazier F, Courcot D, Tall-Dia A, Shirali P (2015). Effects of environmental cadmium and lead exposure on adults neighboring a discharge: evidences of adverse health effects. Environ Pollut.

[6] Li R, Zhou Y, Wang L, Ren G, Zou E (2014). Effects of cadmium alone and in combination with low molecular weight chitosan on metallothionein, glutathione- S -transferase, acid phosphatase, and ATPase of freshwater crab Sinopotamon yangtsekiense. Environ Toxicol.

[7] Lobnik F (2012). Concentration of cadmium in vegetables grown on contaminated gardens and purchesed vegetables. Vet Rec.

[8] Lin Y, Huang JJ, Dahms HU, Zhen JJ, Ying XP (2017). Cell damage and apoptosis in the hepatopancreas of Eriocheir sinensis induced by cadmium. Aquat Toxicol.

[9] Schwerdtle T, Ebert F, Thuy C, Richter C, Mullenders LH, Hartwig A (2010). Genotoxicity of soluble and particulate cadmium compounds: impact on oxidative DNA damage and nucleotide excision repair. Chem Res Toxicol.

[10] Toth R, Scherer D, Kelemen LE, Risch A, Hazra A, Balavarca Y, Issa JJ, Moreno V, Eeles RA, Ogino S, et al. Genetic Variants in Epigenetic Pathways and Risks of Multiple Cancers in the GAME-ON Consortium. Cancer epidemiology, biomarkers & prevention: a publication of the American Association for Cancer Research, cosponsored by the American Society of Preventive Oncology. 2017;26(6):816-825. 10.1158/1055-9965.EPI-16-0728 PMC6054308 28115406

[11] Hossain MB, Vahter M, Concha G, Broberg K (2012). Low-level environmental cadmium exposure is associated with DNA hypomethylation in Argentinean women. Environ Health Perspect.

[12] Pierron F, Baillon L, Sow M, Gotreau S, Gonzalez P (2014). Effect of low-dose cadmium exposure on DNA methylation in the endangered European eel. Environ Sci Technol.

[13] Singh PB, Miller JR, Pearce J, Kothary R, Burton RD, Paro R, James TC, Gaunt SJ (2011). A sequence motif found in a Drosophila heterochromatin protein is conserved in animals and plants. Nucleic Acids Res.

[14] Takebayashi K, Takahashi S, Yokota C, Tsuda H, Nakanishi S, Asashima M, Kageyama R (2014). Conversion of ectoderm into a neural fate by ATH-3, a vertebrate basic helix-loop-helix gene homologous to Drosophila proneural gene atonal. EMBO J.

[15] Phillips AM, Salkoff LB, Kelly LE (2010). A neural gene from Drosophila melanogaster with homology to vertebrate and invertebrate glutamate decarboxylases. J Neurochem.

[16] Singari S, Javeed N, Tardi NJ, Marada S, Carlson JC, Kirk S, Thorn JM, Edwards KA (2014). Inducible protein traps with dominant phenotypes for functional analysis of the Drosophila genome. Genetics.

[17] Gavery MR, Roberts SB (2011). Investigating the role of DNA methylation as an epigenetic mechanism in the pacific oyster (Crassostrea gigas). J Shellfish Res.

[18] Calicchio Rosamaria, Doridot Ludivine, Miralles Francisco, Mehats Celine, Vaiman Daniel (2014). DNA Methylation, An Epigenetic Mode of Gene Expression Regulation in Reproductive Science. Current Pharmaceutical Design.

[19] Field LM, Lyko F, Mandrioli M, Prantera G (2010). DNA methylation in insects. Insect Mol Biol.

[20] Chestnut BA, Chang Q, Price A, Lesuisse C, Wong M, Martin LJ (2011). Epigenetic regulation of motor neuron cell death through DNA methylation. J Neurosci.

[21] Greco CM, Kunderfranco P, Rubino M, Larcher V, Carullo P, Anselmo A, Kurz K, Carell T, Angius A, Latronico MVG (2016). DNA hydroxymethylation controls cardiomyocyte gene expression in development and hypertrophy. Nat Commun.

[22] Szyf M (2011). The early-life social environment and DNA methylation. Clin Genet.

[23] Garg R, Chevala VN, Shankar R, Jain M (2015). Divergent DNA methylation patterns associated with gene expression in rice cultivars with contrasting drought and salinity stress response. Sci Rep.

[24] Song Y, Ci D, Tian M, Zhang D (2015). Stable methylation of a non-coding RNA gene regulates gene expression in response to abiotic stress in Populus simonii. J Exp Bot.

[25] Waters AJ, Makarevitch I, Eichten SR, Swanson-Wagner RA, Yeh CT, Xu W, Schnable PS, Vaughn MW, Gehring M, Springer NM (2011). Parent-of-origin effects on gene expression and DNA methylation in the maize endosperm. Plant Cell.

[26] Lukowiak K, Heckler B, Bennett TE, Schriner EK, Wyrick K, Jewett C, Todd RP, Sorg BA (2014). Enhanced memory persistence is blocked by a DNA methyltransferase inhibitor in the snail Lymnaea stagnalis. J Exp Biol.

[27] Centomani I, Sgobba A, D’Addabbo P, Dipierro N, Paradiso A, Gara LD, Dipierro S, Viggiano L, Pinto MCD (2015). Involvement of DNA methylation in the control of cell growth during heat stress in tobacco BY-2 cells. Protoplasma.

[28] Hongxing Y, Fang C, Chenjiang Y, Jie C, Genfeng Z, Lei W, Yu Z, Ji Q, Hong M (2015). Whole-genome DNA methylation patterns and complex associations with gene structure and expression during flower development in Arabidopsis. Plant J.

[29] Lee ST, Xiao Y, Muench MO, Xiao J, Fomin ME, Wiencke JK, Zheng S, Dou X, De Smith A, Chokkalingam A (2012). A global DNA methylation and gene expression analysis of early human B-cell development reveals a demethylation signature and transcription factor network. Nucleic Acids Res.

[30] Xue D, Jiang H, Deng X, Zhang X, Wang H, Xu X, Hu J, Zeng D, Guo L, Qian Q (2014). Comparative proteomic analysis provides new insights into cadmium accumulation in rice grain under cadmium stress. J Hazard Mater.

[31] Hao J, Liu Y, Xu J, Wang W, Yan W, He A, Fan Q, Guo X, Zhang F (2017). Genome-wide DNA methylation profile analysis identifies differentially methylated loci associated with ankylosis spondylitis. Arthritis Res Ther.

[32] Wang B, Gan Z, Wang Z, Yu D, Lin Z, Lu Y, Wu Z, Jian J (2017). Integrated analysis neurimmiRs of tilapia (Oreochromis niloticus) involved in immune response to Streptococcus agalactiae, a pathogen causing meningoencephalitis in teleosts. Fish Shellfish Immunol.

[33] Yang Qiangzhen, Li Peifei, Wen Yi, Li Sisi, Chen Jun, Liu Xurui, Wang Lirui, Li Xinhong (2018). Cadmium inhibits lysine acetylation and succinylation inducing testicular injury of mouse during development. Toxicology Letters.

[34] Wang L, Li Y, Fu J, Zhen L, Na Z, Yang Q, Li S, Li X (2016). Cadmium inhibits mouse sperm motility through inducing tyrosine phosphorylation in a specific subset of proteins. Reprod Toxicol.

[35] Nguyen KC, Willmore WG, Tayabali AF (2013). Cadmium telluride quantum dots cause oxidative stress leading to extrinsic and intrinsic apoptosis in hepatocellular carcinoma HepG2 cells. Toxicology.

[36] Mw VDD, Murk AJ, Kok DE, Steegenga WT (2016). Comprehensive DNA methylation and gene expression profiling in differentiating human adipocytes. J Cell Biochem.

[37] Tina BM, Mayne BT, Sam B, James B, Rodriguez LCM, Roberts CT (2016). Recent progress towards understanding the role of DNA methylation in human placental development. Reproduction.

[38] Nakashima H, Kimura T, Kaga Y, Nakatani T, Seki Y, Nakamura T, Nakano T (2013). Effects of dppa3 on DNA methylation dynamics during primordial germ cell development in mice. Biol Reprod.

[39] Vizoso M, Puig M, Carmona FJ, Maqueda M, Velásquez A, Gómez A, Labernadie A, Lugo R, Gabasa M, Rigatbrugarolas LG (2015). Aberrant DNA methylation in non-small cell lung cancer-associated fibroblasts. Carcinogenesis.

[40] Loriot A, Reister S, Gk LP, De-Smet C (2010). DNA methylation-associated repression of cancer-germline genes in human embryonic and adult stem cells. Stem Cells.

[41] Clément Bernard, Lamonica Dominique (2017). Fate, toxicity and bioconcentration of cadmium on Pseudokirchneriella subcapitata and Lemna minor in mid-term single tests. Ecotoxicology.

[42] Nair AR, Lee WK, Smeets K, Swennen Q, Sanchez A, Thévenod F, Cuypers A (2015). Glutathione and mitochondria determine acute defense responses and adaptive processes in cadmium-induced oxidative stress and toxicity of the kidney. Arch Toxicol.

[43] Hu F, Liu H, Xie X, Mei J, Wang M (2016). Activated Cdc42-associated kinase is up-regulated in non-small-cell lung cancer and necessary for FGFR-mediated AKT activation. Mol Carcinog.

[44] Chen QY, Jiao DM, Yao QH, Yan J, Song J, Chen FY, Lu GH, Zhou JY (2012). Expression analysis of Cdc42 in lung cancer and modulation of its expression by curcumin in lung cancer cell lines. Int J Oncol.

[45] Garrard SM, Capaldo CT, Gao L, Rosen MK, Macara IG, Tomchick DR (2014). Structure of Cdc42 in a complex with the GTPase-binding domain of the cell polarity protein, Par6. EMBO J.

[46] Harada Y, Tanaka Y, Terasawa M, Pieczyk M, Habiro K, Katakai T, Hanawa-Suetsugu K, Kukimoto-Niino M, Nishizaki T, Shirouzu M (2012). DOCK8 is a Cdc42 activator critical for interstitial dendritic cell migration during immune responses. Blood.

[47] Tatebayashi K, Yamamoto K, Tanaka K, Tomida T, Maruoka T, Kasukawa E, Saito H (2014). Adaptor functions of Cdc42, Ste50, and Sho1 in the yeast osmoregulatory HOG MAPK pathway. EMBO J.

[48] Castel H, Diallo M, Chatenet D, Leprince J, Desrues L, Schouft MT, Fontaine M, Dubessy C, Lihrmann I, Scalbert E (2010). Biochemical and functional characterization of high-affinity urotensinII receptors in rat cortical astrocytes. J Neurochem.

[49] Wang W, Ma L, Becher H, Garcia S, Kovarikova A, Leitch IJ, Leitch AR, Kovarik A (2016). Astonishing 35S rDNA diversity in the gymnosperm species Cycas revoluta Thunb. Chromosoma.

[50] Singh NK, Parmar A, Sonani RR, Madamwar D (2012). Isolation, identification and characterization of novel thermotolerant Oscillatoria sp. N9DM: change in pigmentation profile in response to temperature. Process Biochem.

[51] Cotton LA, Rahman MA, Ng C, Le AQ, Milloy MJ, Mo T, Brumme ZL (2012). HLA class I sequence-based typing using DNA recovered from frozen plasma. J Immunol Methods.

[52] Du J, Zhang L (2015). Integrated analysis of DNA methylation and microRNA regulation of the lung adenocarcinoma transcriptome. Oncol Rep.

[53] Lei Xiaoning, Muscat Joshua E., Zhang Bo, Sha Xuyang, Xiu Guangli (2018). Differentially DNA methylation changes induced in vitro by traffic-derived nanoparticulate matter. Toxicology.

