# Supplementary figures and images for: Cadmium-induced genome-wide DNA methylation changes in growth and oxidative metabolism in Drosophila melanogaster
Source: BMC Genomics. 2019 May 9;20:356. doi: 10.1186/s12864-019-5688-z (PMC6507226; doi:10.1186/s12864-019-5688-z)

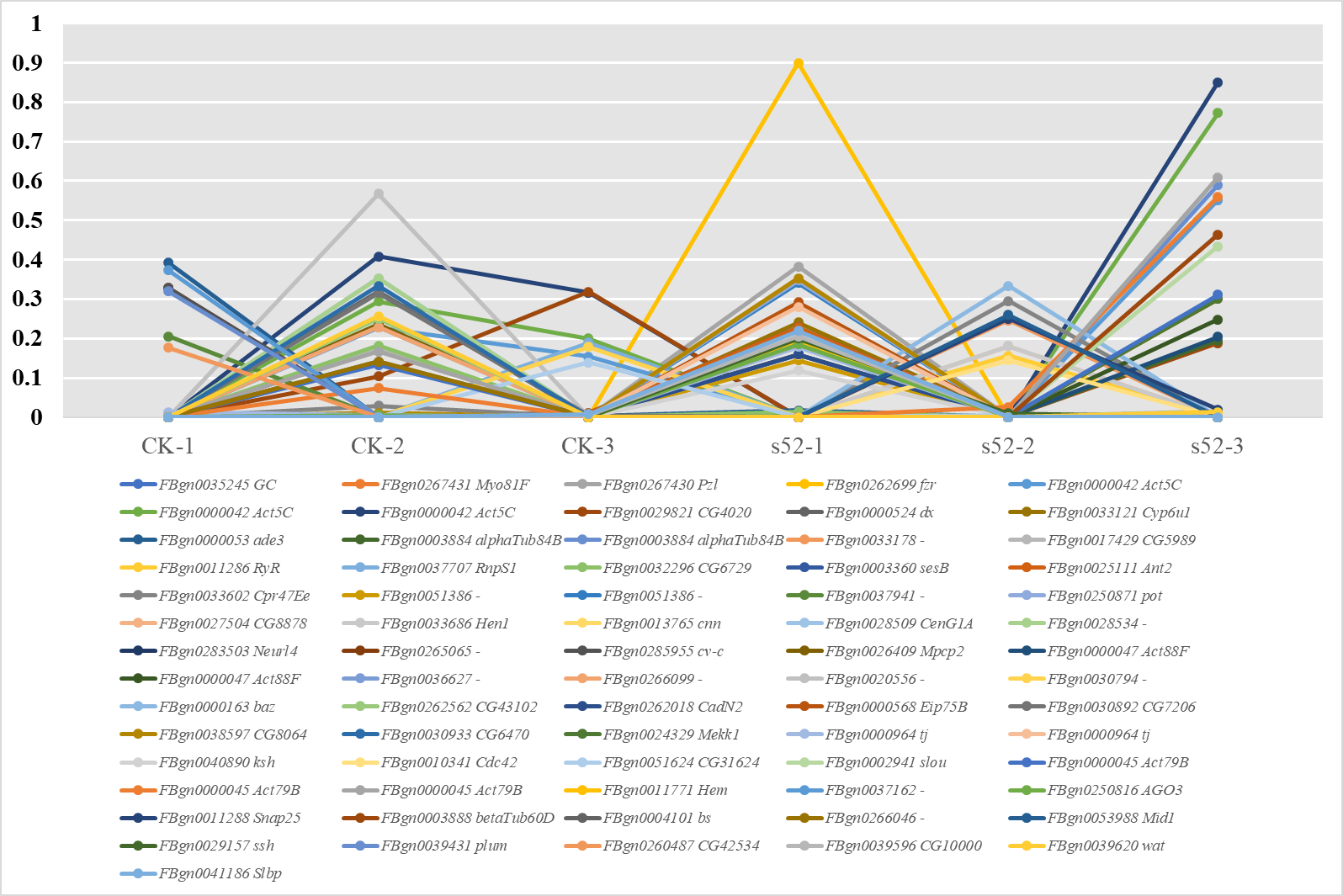

Supplement: Supplementary file 1 — Figure S1. Methylation level of each DMR (all 71 were included) among six samples. The x and y axes show different samples and their overall methylation levels, respectively. (DOCX 312 kb) [file 12864_2019_5688_MOESM1_ESM.docx]

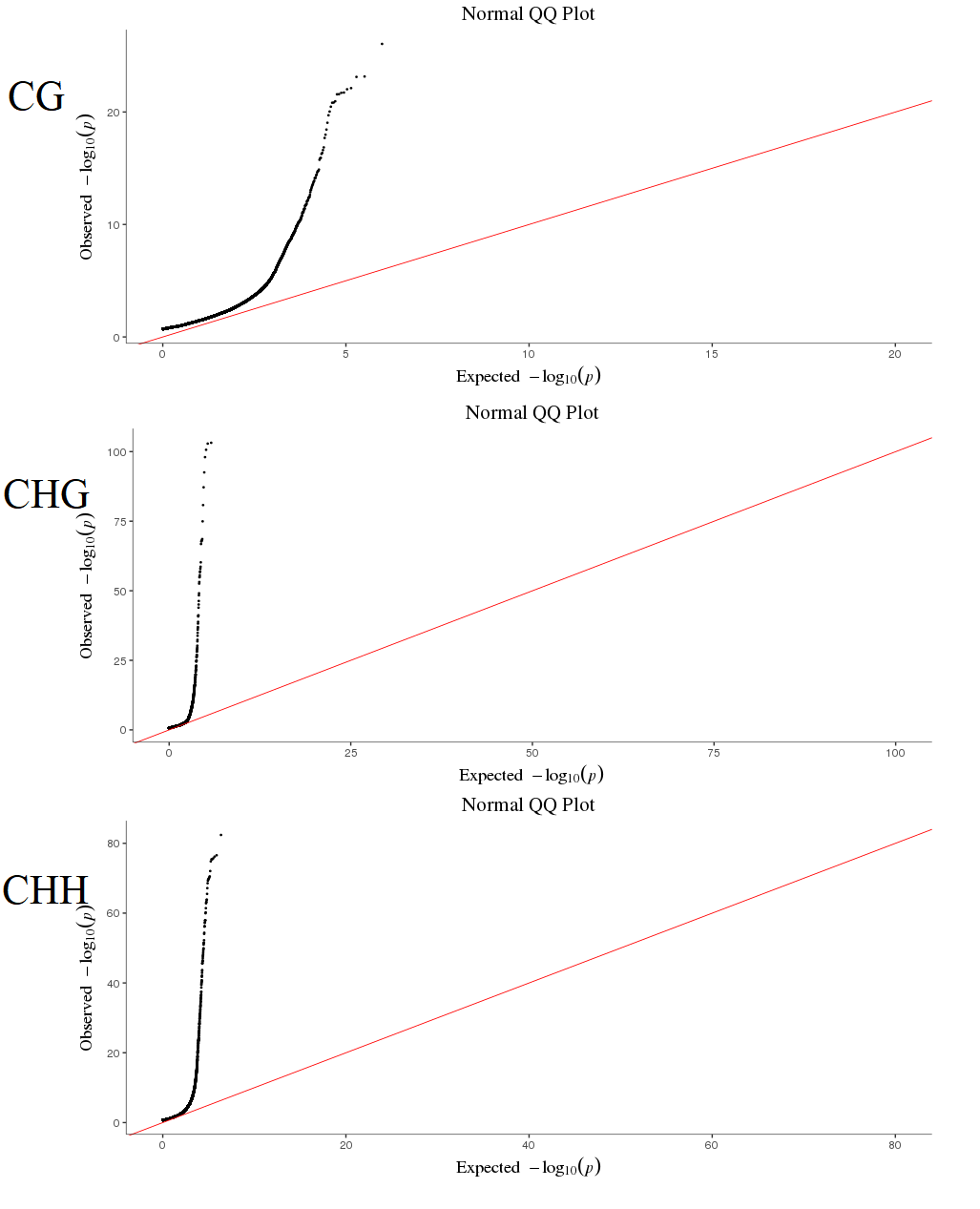

Supplement: Supplementary file 2 — Figure S2. QQ-plots of all the observed log p-values VS the expected log p-values under the null expections of CG (mCG), CHG (mCHG), and CHH (mCHH) sites, respectively. (DOCX 99 kb) [file 12864_2019_5688_MOESM2_ESM.docx]

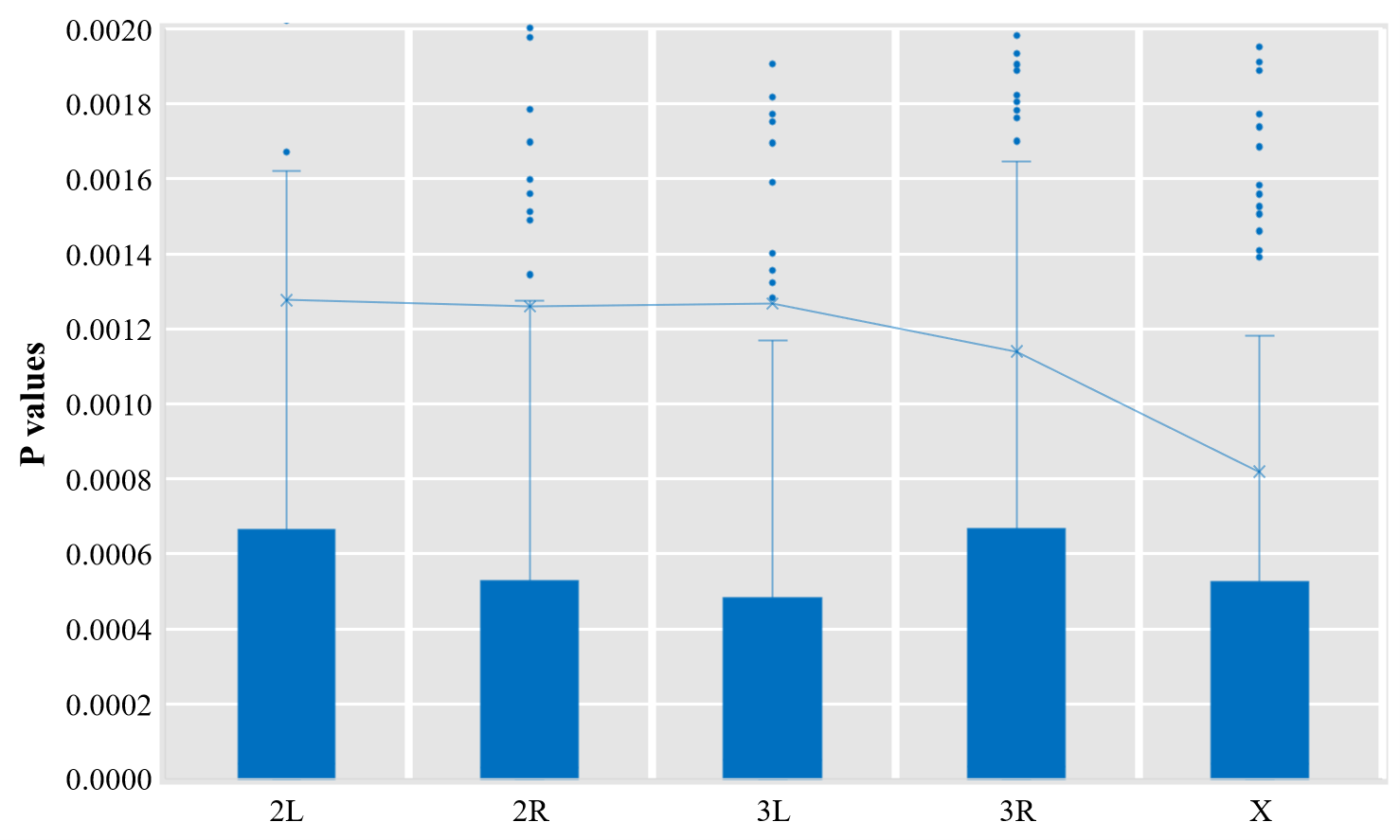

Supplement: Supplementary file 3 — Figure S3. Box plot of P values among all detected methylated nucleotide sites. X and Y axes show different chromosomes and P values, respectively. (DOCX 89 kb) [file 12864_2019_5688_MOESM3_ESM.docx]
